# The impact of perceived parental conflict and school protective factors on chronic stress among Chinese adolescents: a network analysis perspective

**DOI:** 10.3389/fpsyg.2026.1889164

**Published:** 2026-09-18

**Authors:** Shujiao Li, Xiaohui Yu, Zixuan Zhang, Yingying Chi, Yuchen He, Ziying An, Ying Zhang, Lina Li

**Affiliations:** 1 Hebei Key Laboratory of Mental Health and Brain Science, North China University of Science and Technology, Tangshan, China; 2 School of Marxism, Northeastern University at Qinhuangdao, Qinhuangdao, China; 3 School of Resources Engineering, Heilongjiang University of Technology, Jixi, China

**Keywords:** adolescents, chronic stress, network analysis, perceived parental conflict, school climate, teacher support

## Abstract

**Background:**

Chronic stress poses an insidious and cumulative threat to adolescent health. Although family and school environments play pivotal roles, previous research has predominantly examined these domains in isolation. The ways in which familial risks and school protective factors interact to influence chronic stress remain underexplored. To address this gap, this study employs network analysis to elucidate the complex interplay between perceived parental conflict, school protective factors, and chronic stress among Chinese adolescents, thereby providing empirical evidence for multidimensional environmental interventions.

**Methods:**

Employing a combination of convenience and cluster sampling, a cross-sectional survey was conducted among 740 Chinese adolescents (323 males, 43.65%; 417 females, 56.35%; mean age = 16.54 ± 1.15 years). Measurement instruments included the Children's Perception of Interparental Conflict Scale, the Perceived Emotional Support from Teachers Questionnaire, the Perceived School Climate Scale, and the Perceived Stress Scale. Using R version 4.5.2 and the EBICglasso algorithm, a network comprising perceived parental conflict, perceived emotional support from teachers, school climate, and chronic stress was constructed. Node centrality and bridge centrality were computed, followed by accuracy and stability tests.

**Results:**

(1) Teacher caring and teacher support exhibited the highest node strength (1.23 and 1.19, respectively) and expected influence, identifying them as core nodes within the network. (2) Teacher support demonstrated a bridge strength greater than 0.60, serving as a critical bridge between family and school ecosystems. (3) Peer support showed the strongest direct negative partial correlation with chronic stress (β = −0.190). Notably, chronic stress itself displayed low node strength and a predictability of only 27.8%. This indicates that it occupies a peripheral position in the psychological network, manifesting more as a concomitant distal state rather than a core trait driving changes in other variables. (4) A strong positive association bridge emerged among threat, coping self-efficacy, and chronic stress. In the context of perceived parental conflict, the forced mobilization of coping self-efficacy may be accompanied by a simultaneous elevation in chronic stress levels.

**Conclusion:**

Teacher caring and teacher support are associated with critical hubs for family-school collaborative interventions targeting adolescent chronic stress, while peer support is associated with the most direct protective factor. Future interventions should prioritize strengthening teacher and peer support and avoid the blind enhancement of coping self-efficacy in uncontrollable family scenarios, thereby providing a solid empirical foundation for collaborative family-school prevention.

## 1 Introduction

Adolescent mental health problems have become a global public health challenge. According to 2024 data from the World Health Organization, approximately 13.2% of adolescents worldwide aged 10 to 19 suffer from mental disorders (World Health Organization, 2024). As a core variable measuring mental health risk, chronic psychological stress is typically defined as a persistent state of physical and mental tension triggered when an individual perceives long-term external environmental demands as exceeding their coping and adaptive capacities (Lazarus and Folkman, 1984; Torpy et al., 2007). Crucially, consistent with the transactional model of stress, chronic stress is inherently perceptual in nature, it is the individual's subjective appraisal of environmental demands that triggers and maintains the stress response. This perceptual nature makes chronic stress amenable to measurement via self-report instruments that assess individuals' subjective experience of stress in their daily lives. This prolonged state of resource imbalance is particularly severe among adolescents. National epidemiological survey data indicate that, against the backdrop of long-term academic progression pressures, family expectations, and interpersonal competition, Chinese adolescents generally experience high levels of psychological stress. A systematic review and scoping review of psychological stress among Chinese middle and high school students revealed that the incidence of psychological stress symptoms increases significantly with grade level, with the detection rate of stress symptoms reaching up to 40.1% during high school (Li et al., 2024). Furthermore, longitudinal studies on Chinese adolescents indicate that a long-term, high-pressure burden constitutes severe “major chronic stress.” The psychological resource depletion and neurobiological alterations induced by this chronic stress directly lead to the frequent occurrence of internalizing problems such as depression and anxiety in this population (Sheth et al., 2017; Fu et al., 2021). Therefore, exploring the complex interactive relationships between perceived parental conflict and school protective factors within the chronic stress network holds significant practical implications for precisely identifying intervention targets and constructing a well-targeted, collaborative family-school defense mechanism.

Specifically, regarding family factors, adolescents' perceived parental conflict is a critical risk factor for cumulative chronic stress. According to the Cognitive-Contextual Framework, adolescents translate family crises into internal psychological burdens through their assessments of the threat of parental conflict, self-attribution, and coping self-efficacy (Grych and Fincham, 1990). Empirical studies demonstrate that destructive parental conflict not only directly impairs adolescents' psychological adaptation but also drives individuals into a long-term state of chronic stress through persistent threats to their emotional security (Mccauley, 2021; Shelton and Harold, 2008). The three cognitive appraisal dimensions that serve as direct pathways linking perceived parental conflict to psychological maladjustment. Based on the Conservation of Resources Theory, this long-term stress, originating from the family core and difficult for the individual to control, triggers the depletion of deep psychological energy, manifesting as persistent emotional exhaustion and cognitive decline (Hobfoll, 1989; Dong et al., 2025; Li et al., 2025). According to this theory, individuals strive to protect and preserve their valued resources; when these resources are threatened or lost, psychological stress ensues. In the context of parental conflict, adolescents experience a continuous loss of psychological resources, which impedes their ability to recover from stress and contributes to the development of chronic stress. Thus, parental conflict, as an uncontrollable endogenous family stressor, constitutes the deep foundation that compels adolescents into a state of chronic stress and hinders their recovery, an effect achieved through the activation of negative cognitive appraisals and the continuous deprivation of psychological resources. The detrimental effects of family conflict on adolescent adjustment have also been documented in diverse cultural contexts. For instance, Çiçek et al. (2025) found that secure attachment was positively associated with positive childhood experiences, which in turn were linked to lower levels of family conflict and peer bullying among Turkish adolescents, further underscoring the protective role of positive family dynamics across different cultural settings.

Regarding school factors, for adolescents experiencing family adversity such as parental conflict, teacher support, and school protection can compensate for familial risks and buffer the onset of chronic stress. Based on the Stress-Buffering Model of social support, the caring, respect, and emotional support provided by teachers can serve as critical external protective resources, effectively regulating adolescents' cognitive appraisals and stress responses to familial risks (Cohen and Wills, 1985). This model posits that social support protects individuals from the pathogenic effects of stressful events by providing resources that reappraise the threat and enhance coping abilities. Such high-quality teacher-student interactions provide not only substantive coping strategies and emotional reliance but also assist adolescents in rebuilding their psychological security under the dual pressures of academic burdens and family adversity, thereby interrupting the continuous transmission of negative family stress into internal psychological trauma (Butler et al., 2022). According to Ecological Systems Theory, risks within the family microsystem can be compensated by positive factors within the school mesosystem, suggesting that school-based protective factors can buffer the negative effects of family adversity on adolescent development (Bronfenbrenner, 1979). An individual's perception of a positive school climate exerts a healing effect, significantly enhancing psychological resilience (Way et al., 2007; Jia et al., 2009) and providing robust psychological protection for students facing family adversity (Sahib et al., 2025; Xiong et al., 2020). Beyond teacher support, broader social connectedness within the school context has also been shown to buffer the negative impacts of family conflict. Çiçek and Yildirim (2025) demonstrated that social connectedness significantly mediated the relationship between family conflict and depression, anxiety, and sleep problems among Turkish adolescents, suggesting that fostering social connections may serve as a protective mechanism against family-related stressors.

However, although previous research has separately verified the independent impacts of family and school environments, certain limitations remain regarding the integration of these perspectives. First, prior investigations have largely been constrained by the linear assumptions of latent variable models, treating parental conflict or school protection as holistic constructs. This makes it difficult to deeply examine the intricate, mutually triggering mechanisms among specific sub-dimensions, such as conflict intensity and encouraging students. Second, existing empirical studies tend to examine family and school factors in isolation, neglecting their synergistic effects in coping with chronic stress. The ambiguity of core connection nodes across these systems directly limits the scientific formulation of refined, collaborative family-school prevention programs. To address these gaps, the present study employs network analysis to integrate perceived parental conflict, perceived emotional support from teachers, school climate, and chronic stress into a single network structure, aiming to identify core nodes and cross-system bridge pathways within the network.

In summary, this study integrates Chinese adolescents' chronic stress, perceived parental conflict, perceived emotional support from teachers, and school climate into a single network structure, proposing the following hypotheses:

**H1:** In the network of variables related to adolescent psychological stress, perceived emotional support from teachers possesses higher node strength, serving as a core protective node within the network.
**H2:** Teacher support possesses high bridge centrality and may play a role in the pathway from perceived parental conflict to chronic stress.
**H3:** Chronic stress exhibits relatively low node strength and node predictability within the overall psychological network, characterizing it as a resultant, distal variable shaped by the joint action of multiple factors.

Through a highly robust analysis of the network structure, this study aims to identify the most critical intervention targets within the network related to adolescent chronic stress in family and school environments. By revealing the core hubs and buffering pathways within the psychological network, this research ultimately provides empirical support for the construction of a scientific and objective family-school collaborative intervention system.

## 2 Methods

### 2.1 Participants

This study employed a combination of convenience and cluster sampling methods to select 900 middle and high school students for a questionnaire survey. The specific sampling procedure was as follows: First, comprehensively considering geographical location and accessibility, a full-time regular high school in Xinzhou City, Shanxi Province—where the researcher is located—was selected as the target school via convenience sampling. Second, natural classes within the school were designated as the basic sampling clusters. From Grade 10 to Grade 12, 300 students from each grade were selected through simple random sampling for collective test administration. A total of 900 questionnaires were distributed and recovered in this survey. Data collection was conducted in regular classroom settings during school hours. Participants were well-rested and completed the questionnaires voluntarily without time constraints in a comfortable environment. Trained research assistants were present to provide standardized instructions and to address any questions from participants. Students were spaced apart during completion to ensure adequate privacy. All participants were informed that their participation was entirely voluntary and anonymous, that they could withdraw at any time without penalty, and that all data would be kept confidential and used solely for research purposes. To ensure data quality, this study implemented two rigorous independent screening procedures:

First screening (person fit test): Using R software, participants' response patterns were analyzed based on Item Response Theory (IRT) (Liu, 2024). By calculating person fit statistics, participants whose response patterns severely deviated from expectations, exhibited high randomness, or were extremely abnormal were identified and eliminated. Although misfit participants were removed, the representativeness of the remaining sample was tested to ensure the robustness of the conclusions.

Second screening (consecutive identical responses test): Building upon the first screening, long-string analysis was conducted using MATLAB to identify and eliminate invalid samples containing excessively long “consecutive identical responses” in the scales (i.e., participants exhibiting careless or perfunctory responding).

After the aforementioned dual strict quality controls, 740 valid questionnaires were ultimately obtained, yielding an effective response rate of 82.22%. Among the valid sample, there were 323 males (43.65%) and 417 females (56.35%); the average age of the participants was 16.54 ± 1.15 years. Regarding grade distribution, there were 319 students in Grade 10 (43.11%), 161 in Grade 11 (21.76%), and 260 in Grade 12 (35.14%).

### 2.2 Measures

#### 2.2.1 Perceived parental conflict

This study adopted the Children's Perception of Interparental Conflict Scale, originally developed by Grych et al. (1992) and revised by Chi and Xin (2003). The revised scale comprises 38 items covering six dimensions: conflict frequency (CF), conflict intensity (CI), conflict resolution (CR), threat (Threat), coping self-efficacy (CSE), and self-attribution (SA). A 4-point scoring system is utilized, where “1” represents “completely true” and “4” represents “completely false.” A higher score indicates a higher degree of perceived parental conflict by the participant. A sample item is: “My parents often argue with each other in front of me.” In this study, the Cronbach's α coefficient for this questionnaire was 0.835.

#### 2.2.2 Perceived teacher emotional support

This study utilized the Perceived Emotional Support from Teachers Questionnaire for Middle School Students developed by Gao et al. (2017). The questionnaire consists of 18 items encompassing four dimensions: teacher empathy (TE), teacher caring (TC), respect for students (RS), and encouraging students (ES). A 5-point Likert scale is adopted, ranging from 1 (“strongly disagree”) to 5 (“strongly agree”). A higher score indicates that the student perceives a greater amount of emotional support from teachers. A sample item is: “My teacher understands my feelings and emotions.” In this study, the Cronbach's α coefficient for this questionnaire was 0.901.

#### 2.2.3 School climate

This study adopted the Perceived School Climate Scale revised by Jia et al. (2009). The scale contains 25 items covering three dimensions: teacher support (TS), peer support (PS), and autonomy opportunities (AO). The scale utilizes a 4-point scoring system, ranging from 1 (“never”) to 4 (“always”). A higher total score indicates a more positive school climate perceived by the student. A sample item is: “My teachers care about me as a person.” In this study, the Cronbach's α coefficient for this questionnaire was 0.734.

#### 2.2.4 Chronic stress

This study used the Chinese version of the Perceived Stress Scale (PSS), originally developed by Cohen et al. (1983) and revised by Wang et al. (2015), to measure the chronic stress levels of the participants. The scale consists of 10 items, with each item rated on a 5-point scale from 0 (“never”) to 4 (“very often”). The PSS-10 assesses individuals' subjective appraisal of stress in their everyday lives over the past month, capturing the cumulative burden of ongoing daily stressors. The temporal frame of “the past month” reflects sustained stress exposure rather than acute episodes, which aligns with the conceptualization of chronic stress as a persistent state resulting from prolonged environmental demands. The Chinese version of the PSS-10 has demonstrated good reliability and validity (Cronbach's α = 0.91) and is widely used as a self-report measure of perceived chronic stress. The scale score is calculated as the total sum of all items. A higher score reflects a higher level of chronic psychological stress experienced by the individual in daily life. A sample item is: “In the last month, how often have you felt that you were unable to control the important things in your life?” In this study, the score range of the scale was 0–34 points, with an average score of 18.66 points (SD = 4.93).

### 2.3 Statistical analysis and network estimation

This study employed R software for data integration. Initially, descriptive statistics and correlation analyses were conducted for all variables to calculate mean scores and standard deviations. Network analysis was performed using R packages (including qgraph, bootnet, and mgm). The network analysis metrics encompassed network edges, network estimation, network centrality, and predictability, as well as network accuracy and stability. In this study, gender, age, and grade level were included as control variables in the network analysis to account for their potential influence on the estimated associations. All network estimation procedures were performed while controlling for these variables. In this study, 14 dimensions were utilized as network nodes, and the network edges represented the partial correlation coefficients between variables. Network estimation employed the EBICglasso algorithm (tuning parameter γ = 0.5) to obtain a sparse and easily interpretable network structure while controlling the false positive rate. The variance explained (R^2^) for each node was calculated to evaluate the degree to which the node is influenced by the network structure. The study selected four centrality indices to measure the roles of nodes within the network: strength centrality reflects the connective influence of local nodes; closeness centrality reflects the efficiency and independence of information transmission; betweenness centrality measures the degree of control a node possesses as a bridge for information flow; and expected influence reflects the net impact of a node on the overall network. Furthermore, bridge strength was calculated—defined as the sum of connection weights between a node and different communities—to quantify its role in cross-system transmission (Kerriche, 2025). Network accuracy was assessed using the non-parametric bootstrap method to calculate the 95% confidence intervals of the edge weights. Network stability (correlation stability coefficient) was evaluated using the case-dropping bootstrap method, with reference thresholds set at correlation stability coefficient > 0.25 (acceptable) and correlation stability coefficient > 0.50 (ideal) (Epskamp et al., 2018).

## 3 Results

### 3.1 Correlation analysis

This study ultimately included a valid sample of 740 participants. As shown in Table 1, the dimensions of perceived parental conflict generally exhibited significant positive correlations with chronic stress (CS) (*r*: 0.226–0.374, *p* < 0.001), whereas the dimensions of perceived emotional support from teachers and school climate exhibited significant negative correlations with chronic stress (*r*: −0.275 to −0.412, *p* < 0.001). Furthermore, the dimensions of perceived parental conflict also demonstrated significant negative correlations with the majority of dimensions encompassing perceived emotional support from teachers and school climate.

**Table 1:** Correlation analysis results for the full sample.

Variables	*CS*	*TE*	*TC*	*RS*	*ES*	*TS*	*PS*	*AO*	*SA*	*CF*	*CI*	*CR*	*Threat*	*CSE*
*CS*	1													
*TE*	−0.362^***^	1												
*TC*	−0.383^***^	0.800^***^	1											
*RS*	−0.296^***^	0.583^***^	0.691^***^	1										
*ES*	−0.324^***^	0.634^***^	0.722^***^	0.758^***^	1									
*TS*	−0.384^***^	0.596^***^	0.695^***^	0.650^***^	0.708^***^	1								
*PS*	−0.412^***^	0.373^***^	0.412^***^	0.458^***^	0.482^***^	0.586^***^	1							
*AO*	−0.275^***^	0.409^***^	0.494^***^	0.405^***^	0.489^***^	0.613^***^	0.422^***^	1						
*SA*	0.226^***^	−0.096^**^	−0.107^**^	−0.171^***^	−0.112^***^	−0.141^***^	−0.185^***^	−0.011	1					
*CF*	0.258^***^	−0.243^***^	−0.296^***^	−0.250^***^	−0.290^***^	−0.264^***^	−0.294^***^	−0.175^***^	0.328^***^	1				
*CI*	0.262^***^	−0.251^***^	−0.300^***^	−0.261^***^	−0.284^***^	−0.279^***^	−0.309^***^	−0.207^***^	0.302^***^	0.731^***^	1			
*CR*	0.262^***^	−0.241^***^	−0.283^***^	−0.264^***^	−0.290^***^	-−0.310^***^	−0.342^***^	−0.207^***^	0.333^***^	0.649^***^	0.680^***^	1		
*Threat*	0.253^***^	−0.173^***^	−0.188^***^	−0.150^***^	−0.186^***^	−0.185^***^	−0.228^***^	−0.084^*^	0.421^***^	0.449^***^	0.450^***^	0.390^***^	1	
*CSE*	0.374^***^	−0.260^***^	−0.315^***^	−0.252^***^	−0.330^***^	−0.325^***^	−0.313^***^	−0.235^***^	0.438^***^	0.530^***^	0.540^***^	0.530^***^	0.590^***^	1

^*^p < 0.05, ^**^p < 0.01, ^***^p < 0.001.

### 3.2 Network structure estimation and indicator analysis

#### 3.2.1 Network structure of adolescent chronic stress and related variables

The network structure of adolescent chronic stress and related variables is illustrated in Figure 1. From the perspective of the network structure, the nodes of various variable dimensions predominantly exhibit a homologous clustering pattern, forming distinct risk clusters and protective clusters. Perceived emotional support from teachers and school climate form a high-density protective sub-network, with the core connections being teacher caring (TC)—teacher empathy (TE) (β = 0.549) and encouraging students (ES)—respect for students (RS) (β = 0.415). Perceived parental conflict also forms a high-density risk cluster, within which the conflict frequency (CF)—conflict intensity (CI) connection is the strongest (β = 0.437). Cross-construct connections reveal that both chronic stress (CS)—peer support (PS) (β = −0.190) and chronic stress (CS)—coping self-efficacy (CSE) (β = 0.158) satisfy the criterion for high edge weights (*|*β*|* > 0.15). This suggests that a lack of peer support and maladaptive coping self-efficacy are critical direct pathways triggering or maintaining chronic stress, serving as the most crucial bridge edges spanning different psychological ecosystems.

**Figure 1:**
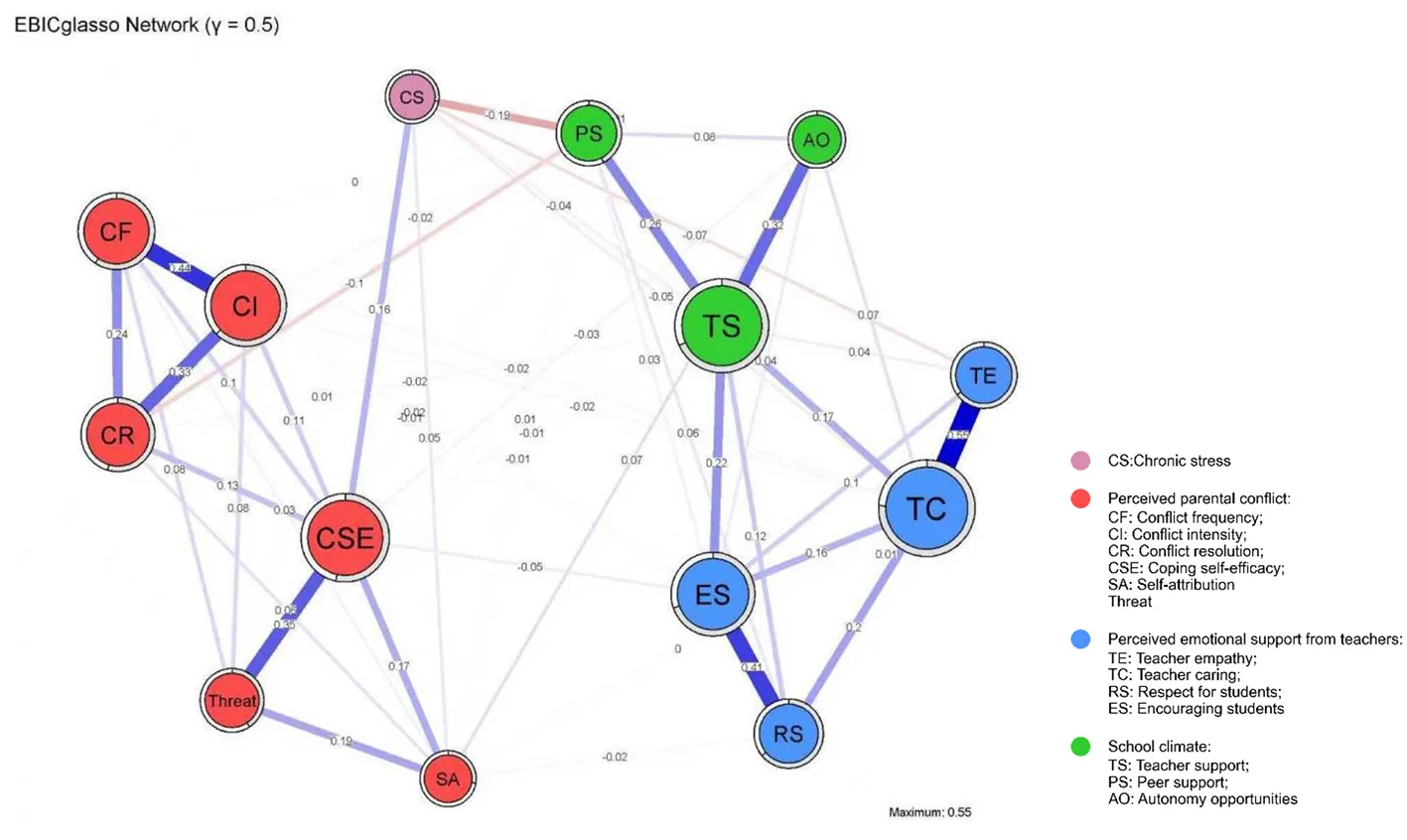
Network structure of chronic stress and related variables among adolescents. CS, Chronic Stress. Perceived parental conflict dimensions: CF, Conflict Frequency; CI, Conflict Intensity; CR, Conflict Resolution; Threat; CSE, Coping Self-Efficacy; SA, Self-Attribution. Perceived teacher emotional support dimensions: TE, Teacher Empathy; TC, Teacher Caring; RS, Respect for Students; ES, Encouraging Students. School climate dimensions: TS, Teacher Support; PS, Peer Support; AO, Autonomy Opportunities. Blue solid lines represent positive partial correlations, and red solid lines represent negative partial correlations. Thicker and darker lines indicate a larger absolute value of the partial correlation coefficient between two nodes, meaning a stronger correlation. The gray portion of the ring surrounding each node represents the predictability (i.e., R^2^) of that node, indicating the proportion of variance in that node that can be explained by other connected nodes in the network. A fuller gray portion indicates a higher degree to which the variable is influenced or explained by other factors in the network.

The pie chart rings surrounding each node in the figure represent the variance explained for that node. The results indicate that the proportion of node variance explained by their adjacent nodes ranges from 27.5% to 75.9%. Among them, teacher caring (TC) demonstrates the highest predictability (*R*^2^ = 0.759), whereas the predictability of chronic stress (CS) is 27.8% (*R*^2^= 0.278), meaning that approximately 27.8% of the variance in chronic stress within the network can be explained by other protective or risk factors.

#### 3.2.2 Centrality indices and stability testing

The scores of the centrality indices (Z-scores) for each dimension are shown in Figure 2. Regarding the indices of strength centrality (Strength) and expected influence (EI), the scores for teacher caring (TC) and teacher support (TS) ranked at the forefront. Peer support (PS) scored relatively high in node strength; however, its expected influence (EI) exhibited a significant decline. Furthermore, for betweenness centrality (Betweenness) and closeness centrality (Closeness), teacher support (TS) demonstrated the highest scores, followed by peer support (PS).

**Figure 2:**
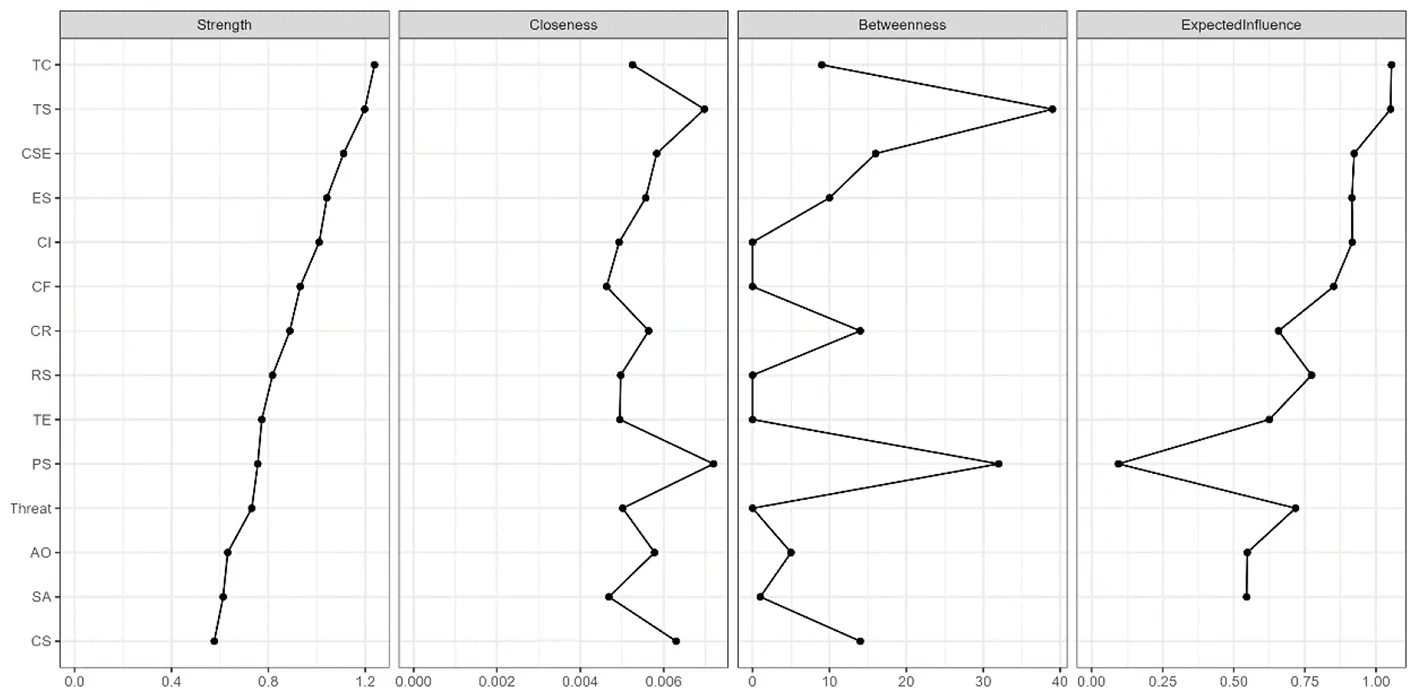
Network node centrality indices (strength, closeness, betweenness, and expected influence).

The bridge centrality (Bridge Strength) index is illustrated in Figure 3. As the figure indicates, teacher support (TS) exhibited the highest bridge strength (>0.60). Simultaneously, chronic stress (CS), acting as an individual's internal psychopathological state, also demonstrated an extremely high bridge strength (approximately 0.58).

**Figure 3:**
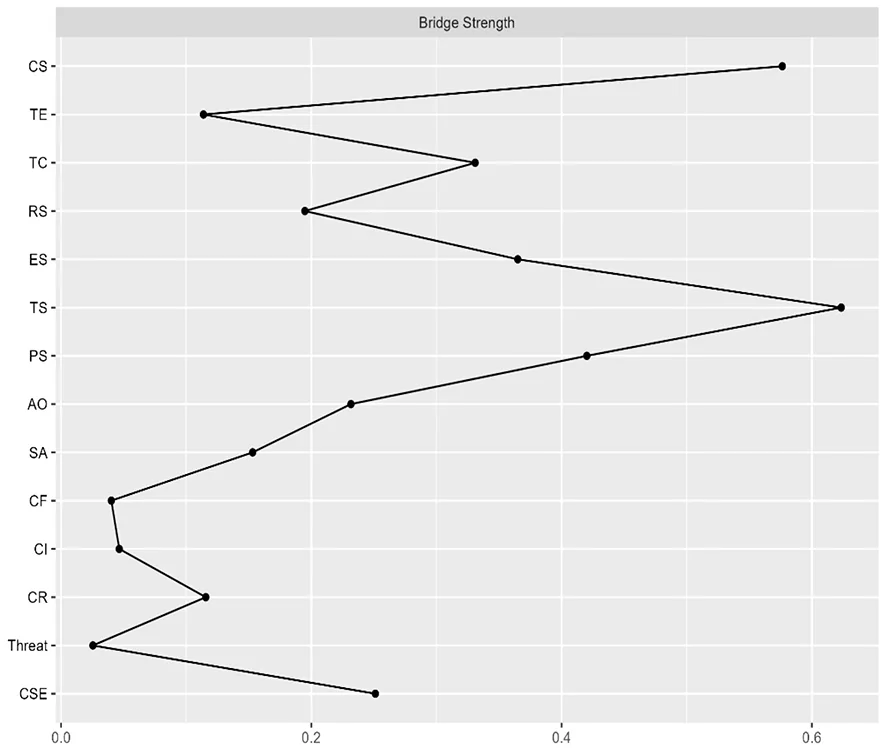
Network bridge centrality indices (bridge strength).

The stability test of the network node centrality indices is shown in Figure 4. The trajectory lines indicate that even after stepwise dropping of up to 75% of the sample cases, the average correlation coefficient between the node strength (Strength) generated by the remaining subsamples and the node strength of the original full sample remains at an extremely high level close to 1.0, showing no precipitous drop.

**Figure 4:**
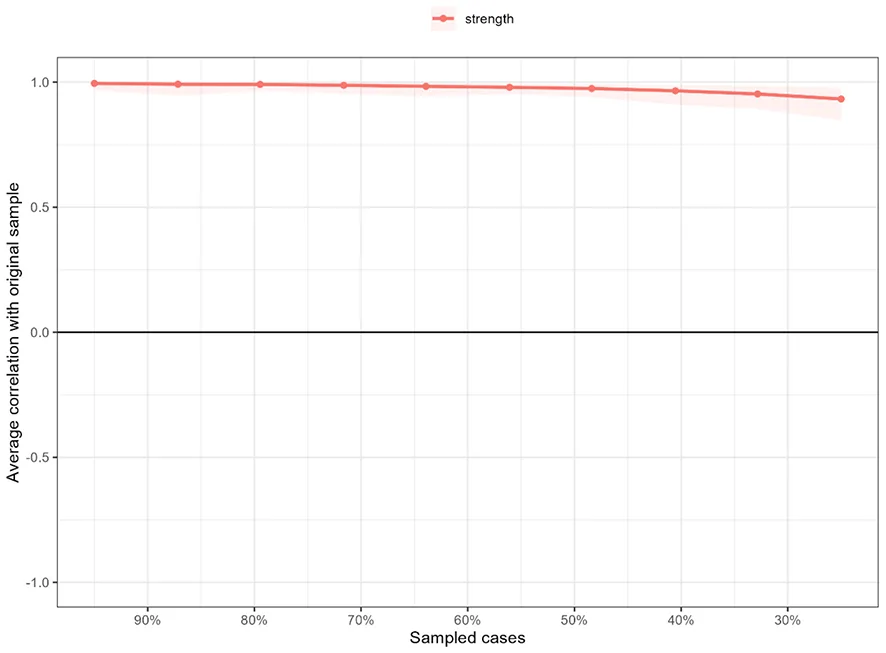
Stability testing of node centrality indices.

Further quantitative calculations reveal that the correlation stability coefficients (CS) for both the network node strength and edge weights reached the maximum testing threshold of 0.75, which is higher than the reference threshold of 0.50 for ideal robustness. This indicates that the network node centrality and bridging characteristics estimated in this study possess excellent statistical stability.

## 4 Discussion

This study integrates adolescents' perceived parental conflict, perceived emotional support from teachers, school climate, and chronic stress into a single network structure. This not only verifies the association of school protective factors (Xu et al., 2025) but also reveals the psychological resource depletion effect of coping self-efficacy within the familial risk system. However, given the cross-sectional design of this study, all interpretations presented below are based on partial correlations identified in the network and should not be interpreted as causal effects. The directional pathways among these variables require further verification through longitudinal or experimental research.

Regarding the core characteristics of the network, teacher support (TS) exhibits an extremely high node strength (1.20) and the highest bridge strength within the network. This demonstrates that teacher support is the core hub connecting the school protective system and the familial risk system, a conclusion consistent with previous research (Zhang et al., 2025; Wang, 2023). The exceptionally high node strength of teacher support suggests that teacher-student relationships serve as a primary source of psychological resources for adolescents, even outweighing peer relationships in terms of overall network influence. Meanwhile, its bridge centrality indicates that teacher support functions as a cross-system connector, showing associations with both the school domain and the family domain. This dual role suggests that teacher support may be a critical intervention target within the network. The node cluster representing perceived parental conflict displays extremely high internal edge density and edge weights, forming a tightly interconnected risk module. This highly correlated local network characteristic suggests that the sub-dimensions of familial risk often co-occur and generate a vicious cycle. Notably, conflict frequency and conflict intensity emerged as the most strongly connected pair within the risk cluster, suggesting that the sheer occurrence and emotional intensity of parental arguments are associated with adolescents' negative appraisals. This implies that interventions aimed at reducing parental conflict should prioritize de-escalating the emotional intensity of arguments rather than merely reducing their frequency. This result aligns with the perspective of Family Systems Theory (Barker and Chang, 2013): when the internal family system becomes dysfunctional, individuals often struggle to obtain the momentum for self-repair within that system and must rely on the intervention of external systems.

Although teacher support serves as the hub center of the network, the study found that peer support has the strongest direct negative edge connected to chronic stress. This finding aligns with the psychological characteristics of attachment shift during adolescence (Markiewicz et al., 2006). According to interpersonal relationship theories, when facing long-term and autonomously irresolvable stressors such as parental conflict, the sense of belonging and emotional resonance provided by peers serve as adolescents' most direct psychological buffers (Rubin et al., 2015). Moreover, peer relationships possess an extremely strong edge-adsorption capacity within stress response networks (Shin and Park, 2025), which may directly relate to lower symptom activation levels (Cheng et al., 2024). The direct negative edge between peer support and chronic stress (β = −0.190) indicates that peer support shows the strongest direct protective association with chronic stress within the network, even stronger than teacher support in terms of direct edges to stress symptoms. This can be explained by the developmental shift during adolescence, where peers increasingly become the primary source of emotional support and social validation, particularly in the context of family adversity where adolescents may seek compensatory belonging from their peer group. This implies that the role of teacher support lies not only in providing direct emotional care and academic guidance but, more importantly, in fostering a harmonious classroom climate and building a positive peer interaction environment to help students secure stable peer support (Farmer et al., 2011). The importance of family relationships in adolescent psychological adjustment extends beyond the domain of family conflict. Yildirim et al. (2025) found that family relationships (including cohesion, expressiveness, and conflict) mediated the association between problematic social media use and psychological adjustment among Turkish adolescents, highlighting the central role of family functioning across multiple risk domains. Consequently, within the complex family-school psychological ecology, this study clearly constructs a dynamic intervention pathway for adolescent chronic stress characterized by “teacher support as the core link and peer support as the frontline buffer.”

The network approach allowed us to identify the most critical intervention targets within the network, providing empirical support for the construction of a scientific and objective family-school collaborative intervention system. Specifically, the high node strength of teacher caring and teacher support identifies them as core protective nodes; the bridge centrality of teacher support suggests its role in connecting family and school systems; and the direct negative association between peer support and chronic stress highlights the most direct protective pathway. These findings collectively demonstrate the relevance of the obtained data to the overarching purpose of this research.

An anomalous positive pathway of threat—coping self-efficacy—chronic stress (Threat-CSE-CS) can be observed from the network structure. The data reveal that coping self-efficacy (CSE) is not only robustly driven by the threat dimension but is also positively correlated with chronic stress. This reflects a counterintuitive phenomenon: under the objective predicament of parental conflict—which individuals cannot change—higher perceived threat impels students to forcibly mobilize psychological resources to cope. From a network perspective, this positive edge between CSE and chronic stress is particularly striking given that CSE is traditionally conceptualized as a protective resource. Its positive association with stress suggests that, within the specific context of parental conflict, the mobilization of coping self-efficacy may function differently than in other stress contexts. According to the Conservation of Resources Theory, forcing minors to resolve family dilemmas that exceed their capabilities leads to severe psychological energy depletion (Hobfoll, 1989). That is, the sustained mobilization of coping efforts in uncontrollable situations may paradoxically accelerate resource depletion rather than alleviate stress. This suggests that the efficacy of coping self-efficacy is context-dependent—it may be protective in controllable situations but potentially detrimental in uncontrollable ones. This is consistent with conclusions from some studies on post-traumatic stress: solely enhancing an individual's coping self-efficacy might be futile and may even increase their cognitive burden, and is associated with the maintenance or elevation of chronic stress levels (Compas et al., 2001; Chen and Miller, 2012). At the practical level, this finding cautions against the blanket enhancement of coping self-efficacy without considering the controllability of the stressor. In school-based mental health programs, interventions for adolescents from high-conflict families might consider focusing on acceptance-based coping strategies, such as emotional regulation and cognitive reappraisal, rather than problem-focused coping strategies that may prove futile in uncontrollable family situations. However, it is crucial to emphasize that this interpretation is speculative and is offered as a hypothesis for future research. Alternative explanations, including reverse causality or the influence of a third variable such as perceived threat, remain equally plausible. Given the cross-sectional nature of our data, we cannot determine the causal direction of this association.

The network analysis shows that self-attribution (SA) occupies a peripheral position in the network and has weak connections to the core symptom chain. One possible interpretation is that the significant development of cognitive abilities during adolescence, aligning with Piaget's stage theory of cognitive development. Furthermore, according to the perspective of Adler's (1931) individual psychology, adolescents have already developed a strong capacity for the separation of tasks, allowing them to objectively differentiate between parental conflicts and their own responsibilities, thereby shaking off the internalized self-blame tendencies highly prevalent during childhood. Distinct from the pervasive “egocentric” attribution characteristic of childhood, adolescents' self-attribution manifests more as a localized stress response rather than a core driver of overall network deterioration (Grych and Fincham, 1990; Fosco and Grych, 2008). From an intervention standpoint, the peripheral position of self-attribution suggests that directing intervention efforts toward modifying adolescents' self-blame may yield limited benefits for reducing chronic stress, as it is not a highly connected or influential node within the network. Instead, interventions might prioritize nodes with higher centrality, such as teacher support and peer support. However, this interpretation is speculative and does not follow directly from our analyses. Future research that includes direct measures of cognitive development and attributional processes is needed to empirically test this hypothesis.

Additionally, chronic stress did not reach the expected central connectivity; its node strength exhibits a minor impact on the network, and its variance explained is relatively low. This indicates that chronic stress is inherently a resultant psychological symptom passively endured by adolescents rather than a pathogenic factor driving changes in other psychological traits (Borsboom, 2017). The low predictability of chronic stress (*R*^2^ = 27.8%) further suggests that chronic stress is influenced by a broader range of factors beyond those captured in the present network, including biological, genetic, and socioeconomic factors not examined in this study. From a network intervention perspective, future interventions could target teacher support (TS) and peer support (PS) at the center of the network to address the negative impacts of family conflict, thereby indirectly relating to alleviation of chronic stress (Li et al., 2025; Wei et al., 2025).

## 5 Conclusion

Based on the network analysis approach, this study constructed a relational network model encompassing chronic stress and variables related to family and school environments, elucidating the complex pathways associated with the formation of chronic stress: teacher support is associated with the core regulatory hub bridging familial risk and school protective systems, peer support is associated with a direct buffering force in alleviating chronic stress, and coping self-efficacy may be associated with a psychological resource depletion effect within the specific context of parental conflict, although this interpretation is speculative and requires further investigation. These findings contribute to the theoretical integration of family and school factors in understanding adolescent chronic stress, demonstrating that network analysis can reveal distinct roles of different protective factors that traditional latent variable models may obscure. Specifically, the identification of teacher support as both a high strength node and a high bridge strength node provides empirical support for ecological systems theory by showing how protective factors across different microsystems can interact to influence adolescent outcomes. Given the cross-sectional nature of the data, these associations should not be interpreted as causal. Future interventions targeting adolescent chronic stress should not be confined solely to the elimination of isolated symptoms or the blind enhancement of individual coping self-efficacy. Instead, they should simultaneously focus on strengthening the emotional support function of teachers and optimizing the quality of peer relationships, while prudently evaluating the cognitive burden potentially imposed by coping self-efficacy, thereby comprehensively improving the mental health status of adolescents. The findings of this study were obtained from a single-school sample and may not be generalizable to all Chinese adolescents. Future research should validate these findings across diverse geographical regions, school stages, and family structures. Building upon the psychological network constructed in this study, future research could conduct longitudinal tracking and empirical intervention studies to further verify the long-term effects of this collaborative family-school intervention pathway and to establish causal directions among the identified associations, ultimately providing more targeted practical guidance for the protection of adolescent mental health.

## 6 Limitations and future study

As an empirical study exploring the protective mechanisms of adolescent mental health, this research holds significant methodological importance. In recent years, network analysis has been widely applied in fields such as psychology, neuroscience, and sociology as an emerging method for investigating complex multivariable systems. Breaking through the limitations of traditional statistical models, this paradigm conceptualizes psychological phenomena as dynamic systems where in core elements (nodes) and their interactions (edges) are directly connected under the influence of the external environment and individual cognition (Borsboom, 2017). Relying on this perspective, the present study integrates familial risks (parental conflict), school protective factors, and chronic stress symptom clusters into a single network for holistic examination. This approach aims to precisely identify the core nodes and cross-boundary bridge pathways within the network, thereby providing more intuitive empirical evidence for collaborative family-school interventions.

Despite the aforementioned theoretical and methodological advantages, this study has the following limitations that warrant refinement in future research:

First, the cross-sectional research design precludes the inference of causal directionality among variables; although network analysis can reveal partial correlation structures among variables, it struggles to capture the dynamic evolutionary patterns of variables over time.

Second, all research data were derived from student self-reports, which may be susceptible to social desirability bias. However, the comfortable testing environment, voluntary participation, and anonymity assurance helped minimize such bias. Nevertheless, self-report measures remain susceptible to general response tendencies, and future research could incorporate multi-method, multi-source data to provide more robust evidence.

Third, the study concentrated on two core domains—familial risks and school protection—without incorporating potential mediating or moderating variables such as motivational variables and individual cognitive resources. Consequently, it is difficult to comprehensively capture all influential pathways involved in the pathogenesis and progression of chronic stress.

Based on these limitations, future research can delve deeper in the following directions: First, longitudinal network analysis or the experience sampling method (ESM) could be adopted to track the temporal interactions between chronic stress and school protective factors, thereby clarifying the predictive direction of core nodes. Second, future investigations could expand the sample scope to include students from diverse geographical regions, school stages, and family structures to test the cross-group stability and generalizability of the network structure identified herein. Third, positive psychological variables such as intrinsic motivation, extrinsic motivation, and life satisfaction could be introduced to explore their mediating or compensatory mechanisms between teacher support and chronic stress, which would enrich the theoretical model of family-school collaboration. Fourth, targeted intervention studies based on the network analysis results could be conducted to verify whether strengthening perceived emotional support from teachers and peer relationships effectively reduces adolescent chronic stress levels, ultimately providing more direct causal evidence for the collaborative family-school prevention of adolescent chronic stress.

## Data Availability

The raw data supporting the conclusions of this article will be made available by the authors, without undue reservation.
